# Who Reports What? A Comparison of Child and Caregivers´ Reports of Child Trauma Exposure and Associations to Post-Traumatic Stress Symptoms and Functional Impairment in Child and Adolescent Mental Health Clinics

**DOI:** 10.1007/s10802-021-00788-y

**Published:** 2021-02-24

**Authors:** Ane-Marthe Solheim Skar, Tine K. Jensen, Anna Naterstad Harpviken

**Affiliations:** 1 Department of Psychology, University of Oslo, Oslo, Norway; 2 Norwegian Centre for Violence and Traumatic Stress Studies (NKVTS), Oslo, Norway

## Abstract

Identifying trauma-related symptoms is important for treatment planning at child and adolescent mental health services (CAMHS), and routine trauma screening may be a first step to ensure appropriate treatment. Studies with community samples have found modest agreement between children’s and caregivers´ report of exposure to potentially traumatizing events (PTEs). However, studies from clinical populations are scarce and the evidence base for screening recommendations is insufficient. The current study explores child and caregiver agreement on the child’s exposure to PTEs and its relationship with the child’s post-traumatic stress symptoms (PTSS) and functional impairment. The sample consist of 6653 caregiver-child dyads referred to Norwegian CAMHS between 2012–2017. The children were 6 to 18 years of age (M = 12.03, SD = 3.14) and 47% were boys and 45% were girls (8% missing). Children reported significantly more exposure to accidents or illness, community violence, and sexual abuse than their caregiver, but there were no differences for reports of domestic violence. Kappa results were fair to moderate, with the highest agreement rate for reports of sexual abuse, followed by domestic violence, community violence, and lowest agreement for accidents or illnesses. There were higher agreement rates among caregivers and older children, and caregivers and girls. In general, the child had higher PTSS and functional impairment scores when child exposure to PTEs were reported by both the caregiver and the child. Both children and caregivers should be included in trauma screening procedures at CAMHS to collect a more complete picture of the child’s experiences and treatment needs.

## Introduction

Studies show that trauma exposed children are amazingly resilient and that there is considerable recovery the first weeks after experiencing trauma (e.g. Miller-Graff & Howell, 2015). Nevertheless, trauma is a significant risk factor for mental illness among children, including somatic problems, depression, anxiety, alcohol misuse, conduct disorder, self-harm, posttraumatic stress disorder (PTSD), and functional impairment (Alsic et al., 2014; Lewis et al., 2019; McLaughlin et al., 2012). Left untreated, posttraumatic stress symptoms (PTSS) may persist and develop to reach the clinical criteria for PTSD. A review of longitudinal studies of PTSD in children showed that there was little change in symptom recovery after six months, indicating that PTSD may become chronic without treatment (Hiller et al., 2016). Reliable information about a child’s exposure to potentially traumatizing events (PTEs) and associated PTSS is therefore important in order to provide appropriate treatment.

There is reason to believe that there is an under-reporting of PTEs in child and adolescent mental health services (CAMHS). In Norway, for instance, 10% of referrals to CAMHS in 2017 were related to “serious reactions after trauma, crises, or catastrophes” (Directorate of Health, 2018). After implementing routine screening of all newly referred cases, it was discovered that in 79% of 10,157 screened cases, exposure to PTEs was reported, pointing to a potential gap between the number of referrals related to PTEs and the actual number of children potentially in need of trauma focused treatment (Skar et al., 2019). Knowledge of trauma exposure is a prerequisite for assessing PTSS, and since children frequently do not report their traumatic experiences, trauma screening at CAMHS is recommended as a first step to identifying trauma and treatment needs (Berliner et al., 2020). The question then remains who we should rely on to report on a child’s exposure to PTEs.

Many clinicians may rely on caregiver reporting rather than the child’s reporting because they believe that caregivers provide more accurate accounts (Grills & Ollendick, 2003). Nevertheless, studies tend to find a discrepancy between children’s and caregivers´ reporting on the extent and type of PTEs the child has been exposed to. Children generally report higher rates of trauma exposure than their caregivers. This is true both for overall trauma exposure, and in cases of violence in particular, where the results have been replicated in both community samples (e.g. Howard et al., 1999; Kuo et al., 2000; Tingskull et al., 2013), at-risk samples (Ceballo et al., 2001; Lewis et al., 2012; Rajan et al., 2014), various pediatric samples including child advocacy centers (Oransky et al., 2013), medical specialty care clinics (Shemesh et al., 2005), and in a trauma-exposed sample receiving follow-up services at a trauma center (Stover et al., 2010). A few studies found that caregivers tend to report significantly higher rates of exposure to accidents (Oransky et al., 2013; Rajan et al., 2014). One study found that children reported community violence more often than caregivers, while caregivers reported more PTEs at home (Thomson et al., 2002). In general, studies find higher agreement of child exposure to PTEs among caregivers and younger children compared to caregivers and older children (e.g. Ceballo et al., 2001; Goodman et al., 2010; Howard et al., 1999; Thomson et al., 2002) and among caregivers and girls compared to caregivers and boys (e.g. Howard et al., 1999; Ceballo et al., 2001).

Since most of the studies to date are based on non-clinical samples, we know little about caregiver´s versus child´s report of trauma exposure in clinical settings. In one study where participants (n = 323) were recruited from both community and psychiatric samples, researchers found that children reported higher rates of maternal violence than the mothers reported themselves. In addition, a higher number of reports of witnessing family violence was detected when both the child and the mother were asked (Kolko et al., 1996). Another study with a clinical sample of 76 acutely traumatized children who were referred to participate in a randomized trial of a secondary prevention program, also found low concordance on PTE reporting between children and caregivers with caregivers underreporting PTEs (Thomson et al., 2002). This study indicates that disagreement is also common in the acute aftermath of trauma, yet it is a lack of knowledge as to whether this is also the case for children with longer lasting difficulties referred to CAMHS for treatment.

One possible explanation for caregiver underreporting may be that parents feel shameful for not being able to protect their child, for instance in cases of domestic violence. In fact, many mothers try to minimize the effects of domestic violence by suggesting that their child was asleep or outside during the violent episodes (Jaffe et al., 1990). The caregiver might also purposively withhold information if the caregiver was the perpetrator of the violence or abuse, or due to feelings such as shame or stigma if they knew but did not do anything to stop it. Caregivers may also worry that disclosure of PTEs that happen in the home and that they may feel responsible for, can lead to involvement of child protective services. Based on this, one might expect lower concordance for interpersonal traumas happening in the home and higher concordance for community violence and non-interpersonal trauma such as accidents and accidental trauma such as illness and sudden death. On the other hand, another suggested explanation for a caregiver’s underreporting of a child’s exposure is that caregivers are not always aware of what has happened to their child outside their home, such as in some cases of sexual abuse, peer victimization or other experiences happening in the community or school (McElvaney, 2013; Richters & Martinez, 1993).

Studies also generally find significant discrepancies between children’s and caregivers´ reports of the child´s trauma-related symptoms. One study with 313 treatment-seeking children found that caregivers reported higher levels of child PTSS in the children than the children did themselves (Wamser-Nanney & Campbell, 2020). Yet, the majority of studies find that children report higher levels of PTSS than their caregivers (Dyb et al., 2003; Ceballo et al., 2001; Meiser-Stedman et al., 2007; 2008; Schreier et al., 2005). Caregiver and child symptom discrepancy may be due to caregiver’s lack of knowledge about common post-traumatic reactions (Schreier et al., 2005). In child clinical samples, however one may expect caregivers to report more accurately because they acknowledge the problems and are seeking help for their child.

Low child and caregiver concordance on trauma exposure and subsequent symptoms is disturbing since low agreement about trauma exposure is found to be related to higher levels of child PTSS (Ceballo et al., 2001; Oransky et al., 2013), more youth violence and distress, and lower self-esteem and problem-solving abilities (Howard et al., 1999). Symptom discordance has also been found to be related to poorer treatment response (Humphreys et al., 2017). Yet, the generalizability of the studies on caregiver´s versus children´s reports of child exposure to PTEs, caregiver-child agreement on reports of child exposure to PTEs, and the association between agreement and PTSS and functional impairment is unclear, as existing studies are few, with low sample sizes, and because studies on clinical samples referred to CAMHS are virtually non-existent.

By examining a large clinical sample of children and their caregivers, this study aims to contribute with novel insight about caregiver-child agreement on youth exposure to PTEs and the associations between agreement and the child’s PTSS and functional impairment. Such knowledge has the potential to strengthen the development of research supported trauma screening and assessment procedures in clinical settings. The first aim of the current study is to investigate agreement between caregiver and child reports of the child’s PTEs (type of trauma and number) in a clinical sample. The second aim is to investigate caregiver-child agreement on reports of PTEs in relation to reports of PTSS and functional impairment and to look at variations across trauma types and child age and gender. We have the following hypotheses:

### *Hypothesis 1:*

Children will report higher levels of exposure to PTEs than their caregivers.

### *Hypothesis 2:*

There will be fair to moderate agreement between caregiver´s and children´s reports of child exposure to PTEs, with higher agreement for domestic violence and accidental traumas (accidents and illness) than for sexual abuse and community violence.

### *Hypothesis 3:*

There will be higher differences between caregiver´s and older children´s reports on the child´s exposure to PTEs compared to caregivers’ and younger children.

### *Hypothesis 4:*

There will be higher differences between caregiver´s and boy´s reports on the child´s exposure to PTEs compared to caregivers’ and girls.

### *Hypothesis 5:*

Caregiver and child agreement on trauma exposure will be related to lower levels of PTSS in the child.

### *Hypothesis 6:*

Caregiver and child agreement on child trauma exposure will be related to lower levels of functional impairment in the child.

## Methodology

### Participants

Participants include a total of 6653 children and their caregivers referred to Norwegian specialized CAMHS from all parts of Norway. The children were 6 to 18 years of age, with a mean age of 12.03 years (SD = 3.14) of whom 45% (n = 2976) were 6–12 years, 28% (n = 1843) were 13–15 years, and 13% (n = 880) were 16–18 years (14% did not provide information about age). The gender distribution was relatively equal among participants, with 47% boys and 45% girls (8% did not provide information about gender).

### Procedures

Data were collected between 2012–2017 as part of a state-funded national implementation of Trauma-Focused Cognitive Behavioral Therapy (TF-CBT) at 44 of a total of 87 Norwegian CAMHS conducted by the Norwegian Center for Violence and Traumatic Stress Studies (NKVTS). As part of this implementation effort, all therapists (psychologists or clinically trained health care workers) at participating CAMHS took part in a 2-h training in routine trauma and PTSS screening and assessment. The training was conducted by psychologists specialized in child and adolescent mental health and trained as TF-CBT trainers. The screening competency of the therapists during the implementation period were assured through the training of local TF-CBT facilitators and consultation calls with clinic leaders who had a specific responsibility to ensure a trauma-informed screening, as well as through the collection of screening data.

The assigned therapist conducted screening of all newly referred children between 6 and 18 years old during one of the first visits to the clinic, followed by PTSS screening and assessment if PTEs were reported by the child, the caregiver, or both. The children were given privacy from caregivers during the screening when this was deemed possible by the therapist. The families were informed that trauma screening was part of routine screening procedures at the CAMHS. If the clinical assessment deemed TF-CBT to be the best available treatment for the child, the therapist presented the treatment model to the child and her or his family. Consent to treatment were ensured through the procedures at the CAMHS and national regulations which states that youth between 16- and 18-years can consent to treatment on own behalf. For children younger than 16-year, consent is required by caregiver(s).

The project was approved by the Regional Committees for Medical and Health Research Ethics (ref. 2009/2304/REK sør-øst) that follow the Helsinki convention and Norwegian laws regarding research with humans. The data was collected as part of routine screening at clinics that all children were involved in as part of treatment assessment, and since we did not receive data that could be associated with the individual child, it was considered register data that we did not need consent to include in research. The study therefore received exemption to informed consent requirement.

### Measures

*Exposure to potentially traumatizing events* were measured through child and caregiver reporting using a brief 15-item trauma screening checklist defining PTEs according to the DSM-5 definition (The American Psychiatric Association, 2013). It was developed for children between the ages 6 and 18 and is used by mental health services in Norway (Jensen et al., 2014). All items on the checklist can be answered with a yes, no, or pass. The number of different PTEs experienced by the child are added to create a total score.

*Post-traumatic stress symptoms and functional impairment* was measured using the symptom part of the Child and Adolescent Trauma Screen (CATS) (Sachser et al., 2017). The CATS assess the frequency of post-traumatic stress symptoms that have occurred within the last two weeks in children and youth following exposure to at least one potentially traumatizing event. It is based on the diagnostic criteria in the Diagnostic and Statistical Manual of Mental Disorders, Fifth Edition (DSM-5) and consists of 20 symptom items and 5 functional impairment items. The scores on the symptom items are added for a total symptom severity score (ranging from 0 to 60). For functional impairment, respondents are asked to mark yes or no to whether the PTSS interfere with getting along with others, hobbies/fun, school or work, family relationships, and general happiness. The total functional impairment score ranges from 0–5 where a higher score indicates higher functional impairment.

### Analysis

All analyses were conducted in SPSS version 26. The sample was divided by age, with primary school age children (6–12 years old) in one group, middle school children (13–15) in another, and high school children (16–18 years old) in a third group. Items within the same trauma category were drawn together to find the prevalence of exposure to accidental traumas or illness (vehicle or other serious accidents, natural disasters, serious illness, medical trauma, or sudden death of someone close), community violence (witnessed or exposed to physical violence in the community, bullying, war, or terror exposure), domestic violence (witnessed or exposed to physical violence in the home), and sexual abuse (touched on private parts against their will or forced to touch others, forced to take sexual pictures or movies, or raped).

Crosstabs with Pearson Chi-Square tests were performed to investigate differences in child age group and gender across dyadic caregiver-child pairs compared to child-only or caregiver-only reporters. Crosstabulation and McNemar tests were run to explore hypothesis 1 (Table 1). Here, agreement point to agreement to whether trauma exposure is reported by caregivers and children. For hypothesis 2–4, Cohen’s Kappa was used to assess caregiver-child agreement (Table 2). The information is organized as follows: ≤ 0 means “no agreement”, 0.01–0.20 shows “none to slight agreement”, 0.21–0.40 demonstrates “fair agreement”, 0.41–0.60 is “moderate agreement”, 0.61–0.80 represents “substantial agreement”, and 0.81–1.00 is close to perfect agreement (Cohen, 1960). Bootstrapping with 10.000 replications and a 95% confidence interval was calculated for the KAPPA. Only confidence intervals where 0 is not included are reported.

**Table 1:** Caregiver-child reports on the child’s exposure to potentially traumatizing events

	n	Caregiver report PTEs (%)	Child report PTEs (%)	*p*
Trauma total	6653	4743 (71.3)	4762 (71.6)	0.669
6–12 years	2976	636 (66.7)	618 (64.8)	0.473
13–15 years	1843	1415 (76.8)	1421 (77.1)	0.809
16–18 years	880	664 (75.5)	717 (81.5)	<0 .001
Boys	3119	2136 (68.5)	2139 (68.6)	0.948
Girls	3023	2276 (75.3)	2300 (76.1)	0.983
Accidents/illness	6222	3306 (53.1)	3428 (55.1)	0.007
6–12 years	2774	1370 (49.4)	1387 (50.0)	0.589
13–15 years	1764	989 (56.1)	1061 (60.1)	0.003
16–18 years	828	490 (59.2)	536 (64.7)	0.004
Boys	2913	1455 (49.9)	1482 (50.9)	0.402
Girls	2849	1614 (56.7)	1713 (60.1)	0.001
Community violence	6092	2143 (35.2)	2597 (42.6)	<0 .001
6–12 years	2729	765 (28.0)	1023 (37.5)	<0.001
13–15 years	1726	767 (44.4)	815 (47.2)	0.027
16–18 years	802	347 (43.3)	420 (52.4)	<0.001
Boys	2871	917 (31.9)	1198 (41.7)	<0.001
Girls	2773	1080 (38.9)	1225 (44.2)	<0.001
Domestic violence	6326	1305 (20.6)	1339 (21.2)	0.349
6–12 years	2846	595 (20.9)	568 (20.0)	0.277
13–15 years	1762	366 (20.8)	401 (22.8)	0.064
16–18 years	835	187 (22.4)	217 (26.0)	0.022
Boys	2949	596 (20.1)	580 (19.6)	0.358
Girls	2892	611 (21.1)	670 (23.2)	0.014
Sexual abuse	6206	331 (5.3)	527 (8.5)	<0.001
6–12 years	2777	91 (3.3)	109 (3.9)	0.098
13–15 years	1753	136 (7.8)	222 (12.7)	<0.001
16–18 years	827	76 (9.2)	149(18.0)	<0.001
Boys	2903	68 (2.3)	94 (3.2)	0.016
Girls	2849	246 (8.6)	402 (14.1)	<0.001

**Table 2:** Kappa agreement for child and caregiver reports of trauma exposure

	n	Kappa*	Only the caregiver report PTEs (%)	Only the child report PTEs (%)	Both reports PTEs (%)	Neither reports PTEs (%)
Trauma total	6653	0.349	13.1	13.4	58.2	15.3
6 – 12	2976	0.340	14.8	14.0	53.4	17.8
13—15	1843	0.349	11.4	11.7	65.4	11.5
16—18	880	0.368	7.7	13.8	67.7	10.8
Male	3119	0.309	14.8	14.9	53.6	16.6
Female	3023	0.374	11.1	11.9	64.2	12.8
Accidents/illness	6226	0.353	15.1	17.0	38.0	29.8
6 – 12	2774	0.366	15.5	16.1	33.9	34.5
13—15	1764	0.330	14.3	18.4	41.8	25.6
16—18	828	0.377	12.0	17.5	47.2	23.3
Male	2913	0.340	16.0	17.0	33.9	33.1
Female	2849	0.360	13.8	17.3	42.8	26.0
Community violence	6094	0.407	10.5	17.9	24.7	46.9
6 – 12	2729	0.335	10.1	19.5	18.0	52.4
13—15	1726	0.475	11.6	14.4	32.8	41.1
16—18	802	0.423	10.0	19.1	33.3	37.7
Male	2871	0.359	10.2	20.0	21.8	48.1
Female	2773	0.453	10.7	15.9	28.2	45.1
Domestic violence	6329	0.407	9.5	10.1	11.1	69.3
6 – 12	2846	0.383	10.5	9.6	10.4	69.5
13—15	1762	0.439	8.6	10.6	12.2	68.7
16—18	835	0.472	7.9	11.5	14.5	66.1
Male	2959	0.372	10.3	9.7	9.9	70.1
Female	2892	0.440	8.6	10.7	12.5	68.2
Sexual abuse	6210	0.491	1.7	4.9	3.6	89.8
6 – 12	2777	0.450	1.6	2.2	1.7	94.5
13—15	1753	0.512	2.1	7.0	5.7	85.3
16—18	827	0.499	1.6	10.4	7.6	80.4
Male	2903	0.315	1.4	2.3	0.9	95.3
Female	2849	0.431	2.1	7.5	6.6	83.8

^*^All results are significant at a *p* < .001 level

To investigate caregiver and child agreement on trauma exposure and associated levels of PTSS (hypothesis 5, Table 3) and functional impairment in the child (hypothesis 6, Table 5), one-way Analysis of Variance (ANOVAs) were performed. Effect size (eta squared; η2) are reported in Tables 3 and 5 for the ANOVA results, where η2 = 0.01 ~ small, 06 ~ medium, and > 0.14 ~ large effect.

**Table 3:** The relationship between caregiver-child agreement on trauma exposure and associated post-traumatic stress symptoms as determined by one-way ANOVAs

	PTSS when only the caregiver report PTEs n (M, SD)	PTSS when only the child report PTEs n (M, SD)	PTSS when both reports PTEs n (M, SD)	F	*p*	*η2*
Trauma total	46 (9.62, 10.29)	236 (12.68, 12.33)	1902 (18.04, 13.39)	25.12	<0.001	0.023
6–12 years	19 (9.45, 11.59)	87 (6.79, 7.79)	622 (13.29, 11.36)	14.04	<0.001	0.037
13–15 years	12 (12.04, 10.79)	67 (15.10, 13.83)	720 (20.50, 13.58)	6.92	0.001	0.017
16–18 years	6 (11.42, 11.33)	58 (18.42, 12.97)	386 (21.88, 14.04)	3.10	0.046	0.014
Boys	20 (11.78, 11.22)	106 (7.84, 8.56)	704 (13.83, 11.73)	12.94	<0.001	0.030
Girls	22 (8.09, 10.07)	118 (16.63, 13.59)	1100 (20.87, 13.68)	14.09	<0.001	0.022
Accidents/illness	211 (15.27, 12.20)	396 (17.53, 13.71)	1162 (17.98, 13.62)	3.63	0.027	0.004
6–12 years	73 (11.68, 10.96)	136 (11.14, 11.08)	360 (13.29, 11.47)	2.04	0.130	0.007
13–15 years	80 (16.70, 10.91)	153 (21.03, 14.33)	430 (20.67, 13.79)	3.18	0.042	0.010
16–18 years	33 (20.82, 14.33)	81 (22.27, 13.60)	252 (20.98, 14.48)	0.27	0.767	0.001
Boys	87 (13.29, 11.85)	160 (12.80, 12.23)	419 (13.27, 11.25)	0.102	0.903	0.000
Girls	109 (17.24, 12.04)	223 (21.01, 13.91)	679 (20.92, 14.11)	3.46	0.032	0.007
Community violence	194 (14.83, 12.32)	410 (15.86, 12.50)	891 (21.48, 13.81)	36.58	<0.001	0.047
6–12 years	75 (12.79, 10.88)	155 (11.50, 11.03)	234 (16.14, 11.96)	8.20	<0.001	0.034
13–15 years	72 (16.77, 13.03)	131 (18.91, 13.13)	387 (23.17, 13.68)	9.81	<0.001	0.032
16–18 years	35 (16.34, 13.98)	89 (19.10, 11.88)	192 (25.61, 14.40)	11.19	<0.001	0.067
Boys	80 (12.74, 10.76)	171 (11.10, 10.39)	313 (16.71, 12.43)	13.88	<0.001	0.047
Girls	108 (16.42, 13.09)	220 (19.53, 12.80)	529 (24.46, 13.80)	21.72	<0.001	0.048
Domestic violence	190 (16.31, 12.10)	282 (19.01, 14.34)	440 (20.66, 13.27)	7.06	0.001	0.015
6–12 years	84 (11.38, 9.14)	88 (10.48, 9.84)	158 (16.03, 11.87)	9.56	<0.001	0.055
13–15 years	56 (19.23, 11.18)	109 (22.32, 14.49)	156 (23.39, 13.72)	2.00	0.137	0.012
16–18 years	31 (21.26, 14.66)	59 (25.46, 13.82)	94 (24.79, 13.79I	0.994	0.372	0.011
Boys	80 (13.61, 10.21)	95 (12.78, 11.35)	163 (16.57, 12.19)	3.81	0.023	0.022
Girls	99 (18.07, 12.65)	176 (22.45, 14.58)	253 (23.75, 13.26)	6.22	0.002	0.023
Sexual abuse	51 (18.54, 12.39)	188 (24.69, 13.63)	198 (26.86, 13.18)	8.02	<0.001	0.036
6–12 years	18 (14.53, 13.66)	27 (17.39, 12.48)	33 (19.42, 11.66)	0.911	0.407	0.024
13–15 years	23 (18.87, 10.37)	86 (26.51, 13.30)	94 (27.29, 12.26)	4.29	0.015	0.041
16–18 years	9 (24.89, 13.47)	61 (25.57, 13.98)	59 (30.98, 13.26)	2.63	0.076	0.040
Boys	13 (16.15, 12.60)	36 (19.63, 14.40)	21 (21.86, 14.91)	0.64	0.529	0.019
Girls	37 (19.18, 12.50)	143 (25.92, 13.35)	167 (27.54, 12.62)	6.35	0.002	0.036

Post-hoc Scheffe tests were used to investigate the mean difference between caregiver and child reports (Tables 4 and 6). For hypothesis 5–6, agreement point to three different categories of agreement: Caregiver only reports child exposure to PTEs; child only reports exposure to PTEs; or both the caregiver and the child reports exposure to PTEs.

## Results

The total sample consisted of 19,710 cases. Cases where only the child (n = 6173) or only the caregiver (n = 6884) were screened for PTEs were excluded in pairwise comparisons. There was a significant child gender difference when comparing dyadic pairs (caregiver-child) with parent-only or child-only reports on PTEs screening (p < 0.001), with a higher percentage of dyadic responses for boys and caregivers (50%) compared to girls and caregivers (42%). Furthermore, there was a significant child age difference when comparing dyadic pairs with parent-only or child-only reports on PTEs screening (p < 0.001). For the youngest children (6–12 years), both the caregiver and the child were screened in 58% of the cases. For children aged 13–15, both the caregiver and the child were screened in 46% of the cases and for the oldest children (16–18), both the caregiver and the child were screened in 29% of the cases.

### Caregiver-Child Reports on the Child’s Exposure to Potentially Traumatizing Events

Results revealed significant differences between children and caregivers in their reports of the child´s exposure to accidents and illness (p = 0.007), community violence (p < 0.001), and sexual abuse (p < 0.001) with children reporting more trauma experiences than their caregivers did (see Table 1). No significant differences were found for total number of traumas or domestic violence. As can be seen from Table 1, there were generally more disagreement among caregivers and older children compared to caregivers and younger children, with children reporting higher levels across all trauma types.

### Caregiver-Child Agreement on the Child’s Exposure to Potentially Traumatizing Events

Fair agreement was observed for total trauma exposure (κ = 0.35), and fair to moderate agreement was observed for the different types of trauma. The lowest agreement – rated as fair according to Cohen (1960) – was found for accidents and illnesses (κ = 0.35), while the highest level of agreement, moderate, was reported for sexual abuse (κ = 0.49). Moderate agreement was found for both community and domestic violence (κ = 0.41, both) (see Table 2).

For total trauma exposure and accidents and illnesses, agreement was fair across all age groups. Agreement ranged from κ = 0.34 to κ = 0.37 for total trauma exposure, and between κ = 0.33 and κ = 0.38 for accidents and illnesses. For community violence, domestic violence, and sexual abuse, agreement was overall moderate in all age groups, ranging from κ = 0.34 to κ = 0.48 on community violence, from κ = 0.38 to κ = 0.47 on domestic violence, and from κ = 0.45 to κ = 0.50 on sexual abuse. However, some divergences were present, as Kappa scores for exposure to community violence and domestic violence were lower among 5- to 12-year-old children than among older age groups (see Table 2).

Bootstrapping results showed that there was an estimated difference of 0.00 for children in the youngest and middle age group for trauma total, with a confidence interval of 0.05 and 0.07. The confidence interval for the youngest and middle age group for accidents and illness is -0.09 and 0.02 with an estimated difference of 0.04. Lastly for accidents and illness, the confidence interval for the youngest and oldest age group is -0.07 and 0.08 with an estimated difference of 0.01. The confidence interval for older and middle age group for community violence is -0.13 and 0.02. Estimated difference is -0.05. Lasty, for sexual abuse, the confidence interval for middle and oldest age group is -0.12 and -0.09 and estimated difference is -0.01.

Caregiver and child agreement for both male and female youth was fair for total trauma exposure, as well as accidents and illnesses. For total exposure, agreement was κ = 0.33 for males and κ = 0.39 for females, and for accidents and illnesses it was κ = 0.31 for males, and κ = 0.37 for females. Conversely, for community violence, domestic violence, and sexual abuse, males reported fair agreement (κ = 0.36, κ = 0.37, and κ = 0.32 respectively) while females reported moderate agreement (κ = 0.45, κ = 0.44, and κ = 0.43) (see Table 2).

Bootstrapping results showed that the confidence interval for gender differences for trauma total is -0.12 and -0.07. Estimated difference is -0.07. For accidents and illness, the confidence interval for gender differences is -0.07 and -0.03 and estimated difference is -0.02. The confidence interval for gender differences for community violence is -0.12 and -0.05. Estimated difference is -0.09. For domestic violence, the confidence interval for gender differences is -0.13 and -0.01 and estimated difference is -0.07.

### Caregiver-Child Agreement on the Child’s Exposure to Potentially Traumatizing Events and Associations with the Child’s Posttraumatic Stress Symptoms

There was a significant effect of agreement on exposure to PTEs (total) [F(2181, 2) = 25.12, p < 0.001, *η2* = 0.023] on PTSS, with post hoc tests showing a significant effect on PTSS when only the caregiver (p < 0.001) or only the child (p < 0.001) reported trauma compared to when reported by both. Furthermore, there was a significant effect of agreement on exposure to accidents [F(1766, 2) = 3.63, p = 0.027, *η2* = 0.004], with post hoc tests showing a significant effect on PTSS when only the caregiver compared to both reported exposure to PTEs (p = 0.027). There was also a significant effect of agreement on exposure to community violence [F(1492, 2) = 36.58, p < 0.001, *η2* = 0.047] on PTSS, with post hoc tests showing a significant difference in level of PTSS when only the caregiver (p < 0.001) or only the child (p < 0.001) compared to when both the caregiver and the child report exposure. Likewise, there was a significant effect of agreement on exposure to domestic violence [F(909, 2) = 7.06, p = 0.001, *η2* = 0.015] on PTSS, with post hoc tests showing a significant effect on PTSS when only the caregiver reported exposure to domestic violence (p < 0.001) compared to when reported by both. Lastly, there was a significant effect of caregiver-child agreement on exposure to sexual abuse [F(434, 2) = 8.02, p < 0.001, *η2* = 0.036] on PTSS, with post hoc tests demonstrating a significant effect when only the caregiver compared to when only the child (p = 0.014) and when only the caregiver compared to when both (p < 0.001) report sexual abuse (see Table 3 and 4).

**Table 4:** Post hoc tests for significant ANOVA results for posttraumatic stress

	I, J	Mean difference (I-J)	Lower bound (95% confidence interval)	Upper bound (95% confidence interval)	*p*
Trauma total	Only caregiver—only child	-3.07	-8.28	2.15	0.356
	Only caregiver—both	-8.42	-13.25	-3.59	<0.001
	Only child—both	-5.36	-7.59	-3.12	<0.001
6–12	Only caregiver—only child	2.66	-4.17	9.49	0.634
	Only caregiver—both	-3.84	-10.13	2.44	0.326
	Only child—both	-6.50	-9.59	-3.41	<0.001
13–15	Only caregiver—only child	-3.055	-13.48	7.37	0.772
	Only caregiver—both	-8.46	-18.14	1.22	0.101
	Only child—both	-5.40	-9.65	-1.15	0.008
16–18	Only caregiver—only child	-7.01	-21.62	7.61	0.501
	Only caregiver—both	-10.46	-24.48	3.56	0.188
	Only child—both	-3.46	-8.25	1.35	0.211
Boys	Only caregiver—only child	3.94	-2.86	10.73	0.365
	Only caregiver—both	-2.06	-8.38	4.26	0.727
	Only child—both	-5.99	-8.90	-3.09	<0.001
Girls	Only caregiver—only child	-8.54	-16.29	-0.79	0.026
	Only caregiver—both	-12.78	-19.97	-5.60	<0.001
	Only child—both	-4.24	-7.47	-1.01	0.006
Accidents/ illness	Only caregiver—only child	-2.26	-5.08	0.55	0.144
	Only caregiver—both	-2.72	-5.19	-0.24	0.027
	Only child—both	-0.45	-2.37	1.47	0.847
13–15	Only caregiver—only child	-4.33	-8.94	0.28	0.071
	Only caregiver—both	-4.00	-8.03	0.10	0.058
	Only child—both	0.361	-2.78	3.50	0.961
Girls	Only caregiver—only child	-3.76	-7.73	0.21	0.068
	Only caregiver—both	-3.67	-7.18	-0.17	0.037
	Only child—both	0.091	-2.53	2.71	0.996
Community violence	Only caregiver—only child	-1.02	-3.86	1.81	0.674
	Only caregiver—both	-6.65	-9.23	-4.07	<0.001
	Only child—both	-5.62	-7.56	-3.68	<0.001
6–12	Only caregiver—only child	1.29	-2.67	5.25	0.725
	Only caregiver—both	-3.35	-7.09	0.39	0.090
	Only child—both	-4.64	-7.56	-1.73	0.001
13–15	Only caregiver—only child	-2.14	-7.00	2.71	0.557
	Only caregiver—both	-6.40	-10.65	-2.15	0.001
	Only child—both	-4.26	-7.60	-0.91	0.008
16–18	Only caregiver—only child	-2.75	-9.47	3.97	0.602
	Only caregiver—both	-9.27	-15.46	-3.08	0.001
	Only child—both	-6.52	-15.46	-3.08	0.001
Boys	Only caregiver—only child	1.64	-2.23	5.50	0.583
	Only caregiver—both	-4.00	-7.54	-0.40	0.025
	Only child—both	-5.60	-8.31	-2.89	<0.001
Girls	Only caregiver—only child	-3.12	-7.00	0.76	0.144
	Only caregiver—both	-8.04	-11.53	-4.56	<0.001
	Only child—both	3.12	-7.57	-2.28	<0.001
Domestic violence	Only caregiver—only child	-2.69	-5.77	0.38	0.101
	Only caregiver—both	-4.35	-7.19	-1.50	0.001
	Only child—both	-1.65	-4.15	0.85	0.271
6–12	Only caregiver—only child	0.904	-3.11	4.92	0.858
	Only caregiver—both	-4.65	-8.21	-1.10	0.006
	Only child—both	-5.56	-9.06	-2.06	0.001
Boys	Only caregiver—only child	0.828	-3.47	5.12	0.894
	Only caregiver—both	-2.96	-6.82	0.91	0.172
	Only child—both	-3.79	-7.44	-0.13	0.040
Girls	Only caregiver—only child	-4.38	-8.58	-0.19	0.038
	Only caregiver—both	-5.68	-9.64	-1.72	0.002
	Only child—both	-1.29	-4.57	1.98	0.626
Sexual abuse	Only caregiver—only child	-6.15	-11.30	-0.99	0.014
	Only caregiver—both	-8.32	-13.44	-3.20	<0.001
	Only child—both	-2.17	-5.50	1.15	0.277
13–15	Only caregiver—only child	-7.64	-14.89	-0.38	0.036
	Only caregiver—both	-8.42	-15.60	-1.23	0.017
	Only child—both	-0.78	-5.39	3.83	0.916
Girls	Only caregiver—only child	-6.74	-12.60	-0.88	0.019
	Only caregiver—both	-8.36	-14.13	-2.59	0.002
	Only child—both	-1.62	-5.24	1.99	0.545

**Table 5:** The relationship between caregiver-child agreement on trauma exposure and associated functional impairment (FI) as determined by one-way ANOVAs

	FI when only the caregiver report PTEs n (M, SD)	FI when only the child report PTEs n (M, SD)	FI when both reports PTEs n (M, SD)	F	*p*	*η2*
Trauma total	22 (1.81, 1.62)	157 (1.73, 1.78)	1359 (2.26, 1.86)	9.24	<0.001	0.012
6–12 years	6 (1.22, 1.51)	54 (0.70, 1.19)	414 (1.49, 1.66)	5.62	0.004	0.023
13–15 years	7 (2.29, 1.98)	46 (2.02, 1.90)	538 (2.62, 1.83)	2.37	0.094	0.008
16–18 years	4 (0.50, 1.00)	40 (2.68, 1.64)	300 (2.72, 1.78)	3.13	0.045	0.018
Boys	10 (1.60, 1.58)	69 (1.25, 1.62)	477 (1.68, 1.74)	1.86	0.156	0.007
Girls	10 (1.00, 1.76)	80 (2.06, 1.78)	819 (2.63, 1.82)	7.20	0.001	0.016
Accidents/illness	140 (2.08, 1.79)	276 (2.26, 1.86)	838 (72.21, 1.89)	0.39	0.680	0.001
6–12 years	40 (1.60, 1.75)	85 (1.18, 1.52)	251 (1.39, 1.62)	1.05	0.351	0.006
13–15 years	60 (2.25, 1.72)	113 (2.67, 1.86)	317 (2.64, 1.88)	1.23	0.294	0.005
16–18 years	25 (2.71, 1.75)	64 (2.92, 1.69)	196 (2.59, 1.84)	0.84	0.435	0.006
Boys	47 (1.91, 1.87)	107 (1.71, 1.72)	293 (1.53, 1.72)	1.22	0.296	0.005
Girls	84 (2.29, 1.71)	165 (2.61, 1.87)	503 (2.60, 1.87)	1.07	0.342	0.003
Community violence	135 (1.90, 1.82)	286 (1.95, 1.78)	648 (2.69, 1.81)	22.05	<0.001	0.040
6–12 years	47 (1.45, 1.38)	102 (1.06, 1.49)	162 (1.81, 1.70)	7.15	0.001	0.044
13–15 years	53 (2.11, 1.98)	93 (2.69 1.78)	293 (7.01, 1.74)	5.72	0.004	0.026
16–18 years	28 (2.25, 1.96)	69 (2.29, 1.55)	146 (3.18, 1.67)	8.39	< 0.001	0.065
Boys	55 (1.44, 1.72)	120 (1.44, 1.70)	210 (2.00, 1.73)	5.14	0.006	0.026
Girls	76 (2.17, 1.80)	152 (2.35, 1.74)	406 (3.09, 1.72)	15.75	<0.001	0.047
Domestic violence	140 (2.02, 1.72)	196 (2.49, 1.88)	320 (2.51, 1.82)	3.90	0.021	0.012
6–12 years	61 (1.64, 1.60)	54 (1.43, 1.69)	105 (1.71, 1.72)	0.53	0.590	0.005
13–15 years	41 (2.17, 1.73)	81 (2.86, 1.78)	123 (2.84, 1.78)	2.50	0.084	0.020
16–18 years	26 (2.58, 1.84)	45 (3.02, 1.71)	72 (3.17, 1.60)	1.18	0.311	0.017
Boys	55 (1.69, 1.70)	58 (1.48, 1.74)	112 (2.07, 1.77)	2.40	0.093	0.021
Girls	76 (2.18, 1.71)	130 (2.95, 1.73)	194 (2.82, 1.80)	4.91	0.008	0.024
Sexual abuse	36 (2.17, 1.99)	138 (2.67, 1.77)	140 (3.34, 1.53)	9.50	<0.001	0.051
6–12 years	12 (1.67, 2.06)	16 (1.06, 1.12)	17 (2.65, 1.58)	4.12	0.022	0.167
13–15 years	17 (2.24, 1.89)	69 (3.09, 1.73)	70 (3.10, 1.38)	2.17	0.117	0.015
16–18 years	6 (3.00 1.67)	45 (2.58, 1.70)	47 (3.87, 1.51)	7.42	0.001	0.129
Boys	8 (1.75, 1.91)	26 (2.08, 1.78)	12 (2.25, 1.66)	0.19	0.826	0.009
Girls	27 (2.30, 1.94)	108 (2.75, 1.76)	123 (3.40, 1.48)	7.08	0.001	0.053

**Table 6:** Post hoc tests for significant ANOVA results for functional impairment

	I, J	Mean difference (I, J)	Lower bound (95% confidence interval)	Upper bound (95% confidence interval)	*p*
Trauma total	Only caregiver—only child	-0.54	1.57	-0.49	0.432
	Only caregiver—both	1.08	2.05	0.11	0.025
	Only child—both	0.54	0.92	0.16	0.003
6–12	Only caregiver—only child	0.63	-1.07	2.33	0.663
	Only caregiver—both	-0.15	-1.78	1.48	0.974
	Only child—both	-0.78	-1.35	-0.209	0.004
16–18	Only caregiver—only child	-2.18	-4.44	0.09	0.064
	Only caregiver—both	-2.21	-4.39	-0.04	0.045
	Only child—both	-0.04	-0.78	0.59	0.991
Girls	Only caregiver—only child	-1.06	-2.56	0.43	0.221
	Only caregiver—both	-1.62	-3.05	-0.21	0.020
	Only child—both	-0.56	-1.09	-0.04	0.031
Community violence	Only caregiver—only child	-0.05	0.52	-0.41	0.964
	Only caregiver—both	0.78	1.20	0.39	<0.001
	Only child—both	0.73	1.04	0.42	<0.001
6–12	Only caregiver—only child	0.39	-0.30	1.07	0.383
	Only caregiver—both	-0.37	-1.01	0.28	0.377
	Only child—both	-0.76	-1.25	-0.26	0.001
13–15	Only caregiver—only child	-0.57	-1.33	0.18	0.173
	Only caregiver—both	-0.87	-1.53	-0.22	0.005
	Only child—both	-0.30	-0.82	0.22	0.372
16–18	Only caregiver—only child	-0.04	-0.96	0.89	0.994
	Only caregiver—both	-0.92	-1.77	-0.07	0.029
	Only child—both	-0.88	-0.89	0.96	0.002
Boys	Only caregiver—only child	-0.01	-0.68	0.68	1.000
	Only caregiver—both	-0.56	-1.19	0.07	0.094
	Only child—both	-0.56	-1.04	-0.08	0.018
Girls	Only caregiver—only child	-0.18	-0.78	0.42	0.767
	Only caregiver—both	-0.92	-1.45	-0.38	<0.001
	Only child—both	-0.74	-1.14	-0.33	<0.001
Domestic violence	Only caregiver—only child	-0.47	-0.96	-0.02	0.067
	Only caregiver—both	-0.49	-0.94	-0.04	0.029
	Only child—both	-0.02	0.43	-0.38	0.991
Girls	Only caregiver—only child	-0.76	-1.39	-0.14	0.012
	Only caregiver—both	-0.64	-1.23	-0.06	0.028
	Only child—both	-0.12	-0.36	-61	0.831
Sexual abuse	Only caregiver—only child	-0.48	-1.27	0.30	0.312
	Only caregiver—both	-1.17	-1.96	-0.39	0.001
	Only child—both	-0.69	-1.18	-0.18	0.004
6–12	Only caregiver—only child	0.60	-0.93	2.14	0.611
	Only caregiver—both	-0.98	-2.50	0.54	0.271
	Only child—both	-1.58	-2.99	-0.18	0.023
16–18	Only caregiver—only child	0.42	-1.31	2.16	0.833
	Only caregiver—both	-8.30	-2.60	0.86	0.462
	Only child—both	-1.29	-2.13	-0.46	0.001
Girls	Only caregiver—only child	-0.45	-1.33	0.42	0.446
	Only caregiver—both	-1.09	-1.96	-0.23	0.008
	Only child—both	-0.64	-1.18	-0.10	0.015

Child age significantly influenced the difference in PTSS between the three agreement groups. For trauma total, there were significant effects across all ages, for children aged 6–12 [F(725, 2) = 14.04, p < 0.001, *η2* = 0.037], for children aged 13–15 [F(796, 2) = 6.92, p < 0.001, *η2* = 0.017], and for children aged 16–18 [F(447, 2) = 3.10, p = 0.046, *η2* = 0.014]. There were also significant effects of caregiver-child agreement on PTSS across all ages for community violence, for children aged 6–12 [F(462, 2) = 8.20, p < 0.001, *η2* = 0.034], 13–15 [F(587, 2) = 9.81, p < 0.001, *η2* = 0.032], and 16–18 [F(313, 2) = 11.19, p < 0.001, *η2* = 0.067]. For accidents [F(660, 2) = 3.18, p = 0.042, *η2* = 0.010] and sexual abuse [F(200, 2) = 4.29, p = 0.015, *η2* = 0.041] there was a significant effect for children aged 13–15 years. For domestic violence, there was a significant effect for children aged 6–12 years only [F(327, 2) = 9.56, p < 0.001, *η2* = 0.055] (see Table 3). See Table 4 for post hoc tests across the different age groups.

Regarding gender, the results showed significant differences in PTSS between the three agreement groups for girls, both on trauma total [F(1237, 2) = 14.09, p < 0.001, *η2* = 0.022], accidents [F(1008, 2) = 3.46, p = 0.032, *η2* = 0.007], community violence [F(854, 2) = 21.72, p < 0.001, *η2* = 0.048], domestic violence [F(525, 2) = 6.22, p = 0.002, *η2* = 0.023], and sexual abuse [F(344, 2) = 6.35, p = 0.002, *η2* = 0.036]. There were significant effects of caregiver-boy agreement on trauma total [F(827, 2) = 12.94, p < 0.001, *η2* = 0.030], community violence [F(561, 2) = 13.88, p < 0.001, *η2* = 0.047], and domestic violence [F(335, 2) = 3.81, p = 0.023, *η2* = 0.022] (see Table 3). See Table 4 for post hoc tests for girls and boys respectively.

### Caregiver-Child Agreement on the Child’s Exposure to Potentially Traumatizing Events and Associations with Functional Impairment

There was a significant effect of caregiver-child agreement on exposure to PTEs (total) [F(1535, 2) = 9.24, p < 0.001, *η2* = 0.012] on functional impairment, with post hoc tests showing a significant effect on functional impairment when exposure to PTEs was reported by the caregiver only (p = 0.025) or the child only (p = 0.003) compared to when reported by both. Also, there was a significant effect of agreement on exposure to community violence [F(1066, 2) = 22.05, p < 0.001, *η2* = 0.040] and sexual abuse [F(311, 2) = 9.50, p < 0.001, *η2* = 0.051] on functional impairment, with post hoc tests showing significant effects when only the caregiver or only the child reported exposure to community violence (p < 0.001) or sexual abuse (p = 0.004) compared to when reported by both (see Table 5 and 6).

The differences in functional impairment between the three agreement groups differed according to age group. For trauma total, there were significant effects for children aged 6–12 [F(471, 2) = 5.62, p = 0.004, *η2* = 0.023] as well as children aged 16–18 [F(341, 2) = 3.13, p = 0.045, *η2* = 0.018]. There were also significant effects of caregiver-child agreement for sexual abuse for children aged 6–12 [F(42, 2) = 4.12, p = 0.022, *η2* = 0.167] and children aged 16–18 [F(95, 2) = 7.42, p = 0.001, *η2* = 0.129]. Lastly, for community violence, there were significant effects across all age groups, for children aged 6–12 [F(308, 2) = 7.15, p = 0.001, *η2* = 0.044], for children aged 13–15 [F(436, 2) = 5.72, p = 0.004, *η2* = 0.026], as well as children aged 16–18 [F(240, 2) = 8.39, p < 0.001, *η2* = 0.065] (see Table 5). See Table 6 for post hoc tests across the different age groups.

There were significant differences in functional impairment between the three agreement groups for girls, both on trauma total [F(906, 2) = 7.20, p < 0.001, *η2* = 0.016], community violence [F(631, 2) = 15.75, p < 0.001, *η2* = 0.047], domestic violence [F(397, 2) = 4.91, p = 0.008, *η2* = 0.024], and sexual abuse [F(255, 2) = 7.08, p = 0.001, 053]. For boys there was only one significant effect of caregiver-child agreement on functional impairment, namely on reports of community violence [F(384, 2) = 5.14, p =  < 0.006, *η2* = 0.026] (see Table 5). See Table 6 for post hoc tests for girls and boys respectively.

## Discussion

This study investigated agreement between caregivers and children aged 6–18 years in their reports of the child’s exposure to PTEs and associated PTSS and functional impairment based on routine screening at Norwegian CAMHS. Poor cross-informant agreement on youth trauma exposure as well as associated PTSS and functional impairment might not only influence the treatment provided to children who are referred to CAMHS, but also caregivers’ abilities to support the child.

Partly in support of *hypothesis 1*, there were significant differences between child and caregiver reports of the child’s exposure to accidents/illness, community violence, and sexual abuse, with children reporting higher rates than their caregivers. This finding is in align with the majority of the literature on non-clinical samples (Howard et al., 1999; Kuo et al., 2000; Tingskull et al., 2013). However, there were no significant differences in total report of trauma or domestic violence. This is surprising since most studies have showed differences in reports of violence in particular. Yet, several studies find that caregivers are less aligned with their children´s violence experiences happening in the community compared to experiences closer to home, hence the results from the current study support this proximity hypothesis. However, previous studies are conducted with non-clinical samples and it could be that parents included from a mental health care setting in the current study are more aware of the child’s PTE’s and therefor have brought the child to therapy. For all violence types, older children report more trauma exposure than their caregivers whereas there are no significant differences among children aged 6–12 years and their caregivers with the exception of reports of community violence where there are significant reporting differences across all three age groups (6–12, 13–15, and 16–18 years). Girls report significantly more exposure than their caregiver´s on accident/illness, community violence, domestic violence and sexual abuse, whereas boys report significantly more exposure than their caregiver´s on reports of community violence and sexual abuse only.

In contrast to *hypothesis 2, a* general higher (moderate) concordance rate was found for reports of sexual abuse, domestic violence and community violence, while lower concordance was found for reports of accidents and illness (fair). In regard to sexual abuse, the current finding is in line with the findings from Stover et al. (2010) which showed the highest agreement occurring in reports of sexual abuse as compared to other trauma types. As suggested in previous studies (Ceballo et al., 2001; Goodman et al., 2010; Howard et al., 1999; Kuo et al., 2000; Stover et al., 2010), it is possible that caregivers are not aware of violence or accidents happening to the child in the community, which might explain the slightly lower agreement for community violence. One study of inner-city mother–child dyads in the USA found that children reported more exposure to community violence while caregivers reported more domestic violence and violence near home (Thomson et al., 2002). We did not measure the relationship to the perpetrator of the sexual abuse or violence; hence we were not able to test this proximity hypothesis. Furthermore, it might be that children report accidents and illness that were somewhat stressful for them without these events being categorized as a trauma by their caregiver. Our findings may also reflect the clinical nature of the sample, and one could expect lower agreement for violence and sexual abuse in non-clinical settings because the trauma and associated symptoms might be less known by the caregivers.

Contrary to *hypothesis 3*, the results indicate that although agreement is more or less the same across age groups, a general increase in agreement occurs across trauma types as the child gets older. This finding is of interest, as several previous studies report lower agreement with caregivers during adolescence than in earlier age groups. In previous studies, it has been suggested that older children may experience more exposure outside the home environment, potentially leading to underreporting of trauma on the part of caregivers (Ceballo et al., 2001; Goodman et al., 2010; Howard et al., 1999; Kuo et al., 2000; Stover et al., 2010). One possible explanation for the increase in agreement as the child gets older might be that younger children more often report scary experiences that are not interpreted as traumatic experiences by the caregiver. Also, in CAMH settings, the existence of mental health problems is acknowledged, and hence caregivers may be more likely to know about their child’s experiences. Yet, it is possible that younger children underreport experiences that they have not told their caregivers about. Despite this, the Bootstrap estimate of confidence intervals suggests that there are some age differences in the Kappa results.

In regard to gender, and in support of *hypothesis 4*, there was generally a higher agreement represented among caregivers and girls, than among caregivers and boys. It is not clear whether this result, which is consistent with findings from Ceballo et al. (2001) and Howard et al. (1999), might be due to differences in gender roles and expectations which makes caregivers more likely to ask and girls more likely to tell about exposure to PTEs. Further studies investigating caregiver-child concordance on reports of the child´s trauma history should pay attention to the role of child gender and investigate associated factors which might help explain this finding.

In contrast to *hypothesis 5*, children who were in dyads where agreement about exposure was established were more likely to have high levels of PTSS. This finding might indicate that the PTE is more likely to be defined as a traumatic experience both by the child and caregiver, hence the elaborated levels of PTSS. A shared agreement on the PTE could make it easier for the child to talk about their PTSS as the caregiver is aware of the trauma and therefore is aware of the child’s internalized struggles. This means that the child´s PTSS may be more likely to be interpreted as trauma related by the caregiver, which again may be related to CAMH referrals. In the study by Lewis et al. (2012), the child experienced higher levels of PTSS when trauma was reported by both the caregiver and the child or only by the child compared to caregiver-only reports. Furthermore, concordance related to youth-witnessed violence was associated with higher caregiver endorsement for counselling services (Lewis et al., 2012). Bambrah and colleagues found that an increased level of agreement from pre- to post-therapy related to the child´s PTSS was associated with parent and child reported improvements in parent reported child PTSS, internalizing and externalizing problems (Bambrah et al., 2018). This speaks to the importance of trauma informed referral services. Furthermore, caregiver-child concordance could be a vital factor in the recovery process of the child through facilitating social support. In a study with 96 child-caregiver dyads receiving TF-CBT, improvements in discordance during the therapeutic period predicted PTSS reduction (Bambrah et al., 2018). TF-CBT is a family-oriented therapy hence this finding might indicate that caregivers were better able to support the child when they during the therapeutic process received the same understanding of the trauma experience.

In contrast to *hypothesis 6*, high agreement on reports of trauma exposure was related to higher levels of functional impairment in the child. Hence agreement about exposure does not seem to serve as a preventive factor for functional impairment in the child through making the caregiver more able to provide support and safety while assisting the child in developing appropriate coping strategies in the post-trauma period (Kliewer et al., 2004; Ozer, 2005; Stallard et al., 2001). High concordance has previously been related to better child psychological functioning in at-risk samples (Ceballo et al., 2001; Oransky et al., 2013) whereas low concordance has been related to poorer child outcomes (violent behavior, distress, lower self-esteem, lower problem-solving skills) (Howard et al., 1999). In the study by Oransky et al. (2013), children reported significantly higher rates of PTEs than caregivers did, and discrepancies were significantly correlated with higher levels of depression, PTSS and functional impairment.

Through investigating caregiver-child concordance on trauma exposure and the relationship with the child’s PTSS and functional impairment in a nation-wide, large-scale clinical mental health sample, this study provides knowledge that can be used to develop evidence-based screening procedures. The results are in line with findings from community and at-risk samples, demonstrating that children’s trauma exposure is reported differently between caregivers´ and children in various settings. Although utilization of unreliable informants might lead to a type I or type II error, the literature shows that there is no rule on who is the “golden” or “optimal” informant on reports of childhood psychopathology (De Los Reyes & Kazdin, 2005). It therefore stands in good reason to believe that different informants might provide valid and reliable insights in different settings and for different types of information.

One should, however, be aware of possible biases in the current study, corresponding to the four factors assumed by Kraemer et al. (2003) to have the potential to influence reports. First, an important limitation is that we had limited data on *informant characteristics* (Kraemer et al., 2003), including ethnicity, family income, and the responding caregiver’s gender or relationship with the child. The sexual abuse category lacked the specificity of sexual abuse by a family member, other trusted adult, other adolescent, or a stranger, and it might be that the relationship with the perpetrator would have influenced the results. Further research should therefore investigate the child´s relationship with the perpetrator and differentiate incest from other types of relationships with the perpetrator. Furthermore, one might hypothesize that disclosure of traumatic experiences to caregivers is related to a positive caregiver-child relationship in general. For sexual abuse, agreement on exposure may indicate a particularly close relationship, as this type of trauma is often associated with shame and guilt, which makes disclosure difficult for many youths. As caregiver support or conflict could be predictive of PTSS following trauma (e.g. Bokszczanin, 2008), future clinical studies on caregiver-child agreement on trauma and PTSS should include measurement of caregiving and caregiver-child relationships to investigate this hypothesized relationship further. Also, we did not have data on the caregivers´ own mental health, which in previous research has been shown to be related to child PTSS (Morris et al., 2012), and identified as a factor influencing caregiver ratings of their child’s stress symptoms (Kassam-Adams et al., 2006; Oransky et al., 2013; Shemesh et al., 2005).

Second, we did not control for the *context* (Kraemer et al., 2003) in which the screening was conducted, such as whether the child screening was completed in the presence of their caregivers. In particular, the therapeutic context may have affected children´s willingness to disclose trauma histories compared to research contexts where the respondents are promised confidentiality, which might have made adolescents report more consistent with parent reports, especially if the caregiver was present during the screening.

Third, we were not able to control for the perspectives and *possible biases of the informant* (Kraemer et al., 2003), such as the time since the child experienced the PTE. These factors might influence the results, considering previous findings showing a decrease over time in report discrepancy related to PTSS (Dyb et al., 2003; Schreier et al., 2005). Furthermore, we don't know what factors may have accounted for some dyads to have both reports and others to have only a child or a parent report and if any of these factors might be trauma- or reporting-related.

Finally, there might be *measurement errors* (Kraemer et al., 2003). We did not measure caregiver and child reports of different trauma types across different assessment methods, nor did we have information about how the screening was conducted. A study comparing discordance among caregiver-child reports across assessment methods found that caregivers generally reported higher levels of self-exposure to PTEs as well as child PTEs via interview as compared to checklists, with the exception of higher report frequencies of both caregiver and child direct violence exposure through checklists than through interview (Glackin et al., 2019). Based on this, Glackin et al. (2019) suggested that caregivers may feel more comfortable disclosing severe PTE exposure via checklists rather than through interviews.

Despite these possible limitations in the current study, we can assume the data is representative for this population as data were collected from the majority of children referred to half of all the CAMHs in Norway during a 6-year period. Identifying reliable methods to trauma and PTSS screening is significant not only for the individual child and family, but also for society, as untreated traumatic reactions are likely to follow the child into adulthood with consequences affecting academic life, work life, mental and somatic health, and life expectancy (Davidson, 2000; Olff et al., 2019; Priebe et al., 2009; Rothenhäusler, 2006). To provide the most accurate picture of the child’s treatment needs, a clinical implication from this study is that both the caregiver and the child should be included in trauma screening. ​In the current study, there was a higher probability that dyads with boys and caregivers were screened compared to girls and caregivers, as well as a higher probability that dyads of younger children and caregivers were screened than older children and caregivers. This might imply the need for age appropriate screening guidelines which facilitate universal screening in CAMH contexts in order to prevent biases in screening and hence, also the probability to receive trauma focused treatment. Female child gender and younger age seem to influence associated levels of child PTSS and functional agreement. A previous study demonstrated that reporting disagreement had a negative effect on internalizing problems on girls only but on externalizing problems for boys and girls the same (Zimmerman & Farrell, 2013). Younger children are often more dependent upon support by caregivers, underscoring the importance of establishing a shared understanding of exposure among caregivers and younger children in child trauma treatment.

Not surprisingly, there were fewer dyads with both caregiver and child reports among adolescents than younger children. It might be that some adolescents came to the clinic without any caregiver, especially since the screening in some cases might have been conducted at the second meeting at the clinic. Relying on single reports, in general, and solely caregiver reports in particular, is likely to lead to a misdiagnosis and hinder effective trauma focused treatment. Children’s reports of violence and other types of trauma experiences are associated with their wellbeing and function (Kolko et al., 1996; Reigstad et al., 2006), which further emphasizes the importance of asking the child directly about her or his experiences. When developmentally appropriate, therapists should strive to screen children for trauma experiences without the presence of their caregiver in order to facilitate open and honest responses and guide bias against false negative reports. Caregivers, on the other hand, may identify traumas that might not be defined as such by the child, or identify traumas the child does not want to report. Conversely, it is also possible that children report traumatic events that are not perceived as such by the caregiver. Knowledge about these instances are relevant knowledge for a therapist and demonstrates the importance of gathering information about PTEs and associated PTSS and other trauma-related symptoms from multiple sources in a clinical context. Underreporting of PTEs or PTSS by the caregiver might pose an extra burden on the child by hindering adequate treatment and support, and future studies should investigate this further. As such, future, expanded studies on this topic have the potential to inform treatment aimed at mitigating the impact of childhood trauma for children and for adults traumatized during childhood.

## Data Availability

The data are available from the corresponding author on reasonable request.
